# The efficacy and safety of electroacupuncture for women with pure stress urinary incontinence: study protocol for a multicenter randomized controlled trial

**DOI:** 10.1186/1745-6215-14-315

**Published:** 2013-09-30

**Authors:** Zhishun Liu, Huanfang Xu, Yuelai Chen, Liyun He, Jia Liu, Shiyan Yan, Ruosang Du, Jiani Wu, Baoyan Liu

**Affiliations:** 1 Guang’anmen Hospital, China Academy of Chinese Medical Sciences, Beijing 100053, China; 2 Yueyang Hospital of Integrated Traditional Chinese and Western Medicine affiliated to Shanghai University of Traditional Chinese Medicine, Shanghai 200437, China; 3 China Academy of Chinese Medical Sciences, Beijing 100700, China

**Keywords:** Electroacupuncture, Pure stress urinary incontinence, Efficacy, RCT, Study protocol

## Abstract

**Background:**

Although available evidence relating to its effectiveness is weak, acupuncture is used as an alternative therapy for stress urinary incontinence. We report a protocol of a randomized controlled trial using electroacupuncture (the passing of a weak current between inserted acupuncture needles) to treat women with pure stress urinary incontinence.

**Methods/Design:**

This is a large-scale multicenter subject-blinded randomized controlled trial. A total of 500 women with pure stress urinary incontinence will be randomly assigned to two groups: a treatment group and a control group. The treatment group will receive electroacupuncture with deep needling at acupuncture points BL33 and BL35. The control group will receive sham electroacupuncture with non-penetrating needling at sham locations for the acupuncture points of BL33 and BL35. Participants will be given three sessions a week for 6 weeks. A 24-week-long follow-up will be conducted. The primary outcome will be the change in amount of urine leakage at the sixth week from a baseline measured by a 1-h pad test. The secondary outcomes include: the 72-h incontinence episode frequency based on a 72-h bladder diary; the score of International Consultation on Incontinence Questionnaire-Urinary Incontinence Short Form; the degree of urinary incontinence based on a 72-h bladder diary; self-assessment of the therapeutic effect; weekly consumption of pads; application of other treatments for stress urinary incontinence; and subgroup analysis stratified by incontinence severity. The safety of electroacupuncture will also be assessed.

**Discussion:**

This trial will help to identify whether electroacupuncture is effective for stress urinary incontinence, and, if so, whether it is a therapeutic effect rather than a placebo effect.

**Trial Registration:**

Clinical Trials.gov NCT01784172

## Background

Stress urinary incontinence (SUI), defined as the involuntary leakage of urine on effort or exertion, or on sneezing or coughing [1], is a common complaint among adult women. It is generally considered that urinary incontinence (UI) is a prevalent cross-cultural healthcare problem. The global median prevalence of female UI was found to be 27.6% (range: 4.8-58.4%) [2], among which SUI accounted for about 50% [2,3]. Prevalence of SUI increases with age, and a global annual incidence of 4% to 10% was estimated in longitudinal studies [4]. SUI affects not only a patient’s physical health but also her social and psychological wellbeing. Patients are more likely to become depressed and are more prone to self-abasement than other women because of the uncontrollable leakage of urine. This makes them nervous about taking long journeys or participating in social activities. These negative quality-of-life effects are greater than those resulting from some major chronic conditions (diabetes, hyperlipidemia, and chronic kidney disease) [5].

The International Consultation on Urological Diseases (ICUD) recommends lifestyle regulation, behavior therapy, pelvic floor muscle training (PFMT), and functional electrical stimulation as conventional therapies for mild and moderate female SUI. A systematic review supported PFMT as a conservative grade-A recommended therapy for female SUI with a 30-60% effective rate; however, for it to be maximally effective it needs to be practiced for at least 3 months [6]. Additionally, the positive effects of PFMT are closely related with patient compliance [7], which decreases with the extension of training time. PFMT is seldom used in China since skilled physiotherapists are rare. For moderate to severe SUI, a mid urethral sling with surgical mesh is widely used. However, the use of surgical mesh increases adverse events such as pain, infection, dysuria, and neuromuscular problems. A safety communication from the U.S. Food and Drug Administration (FDA) on serious complications associated with transvaginal placement of surgical mesh for pelvic organ prolapse was issued on 13 July 2011 [8]. Several randomized controlled trials (RCTs) showed that acupuncture, by decreasing urine leakage and improving patients’ quality of life, maybe an alternative therapy for SUI [9,10]. However, because of the limited evidence [11], high-quality RCTs are needed to assess the efficacy of acupuncture for treating SUI.

We have designed a multicenter RCT on pure SUI patients that do not exhibit symptoms suggestive of overactive bladder or voiding dysfunction [12]. Using sham electroacupuncture (EA) as control, the trial aims to identify the efficacy of EA by answering two questions: (1) Is EA effective for women with pure SUI? and (2) If effective, is it a therapeutic effect rather than a placebo effect? Additionally, we intend to establish the safety and acceptance of EA in women with pure SUI. The study is registered with a Clinical Trials.gov identifier NCT01784172.

## Methods

### Study design

**Figure 1:**
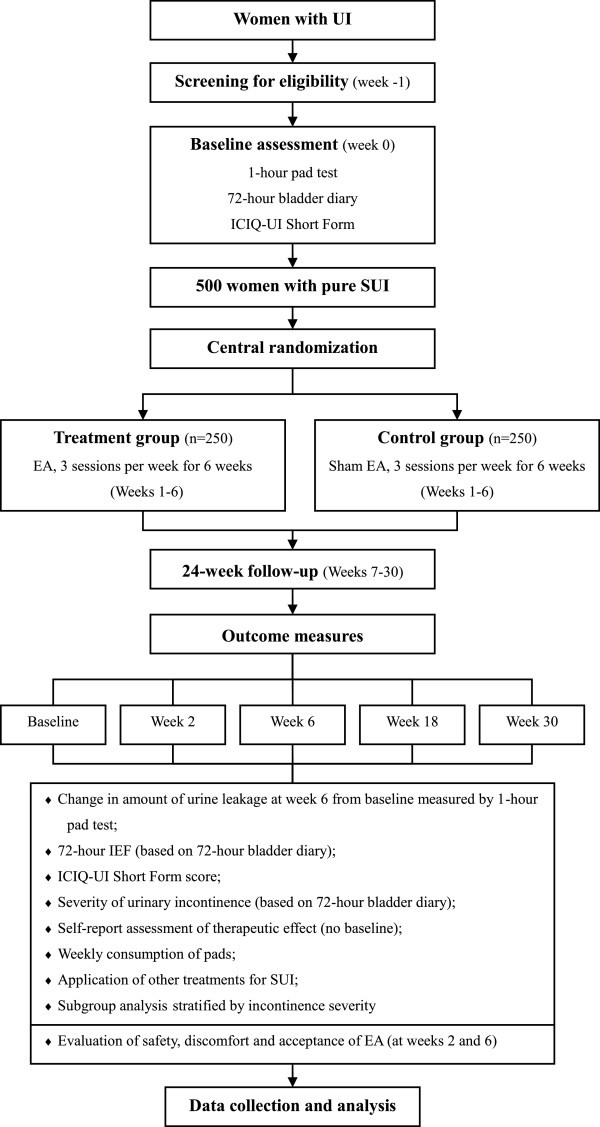
Trial flow chart.

This is a multicenter subject-blinded randomized controlled trial comparing EA with sham EA. Five hundred women with pure SUI will be recruited from the following 12 hospitals in China: Guang’an men Hospital of China Academy of Chinese Medical Sciences (CACMS); Xiyuan Hospital of CACMS; Dongzhimen Hospital affiliated to Beijing University of Chinese Medicine; Yueyang Hospital of Integrated Traditional Chinese and Western Medicine affiliated to Shanghai University of Traditional Chinese Medicine (TCM); West China Hospital of Sichuan University; First Teaching Hospital of Tianjin University of TCM; First Hospital of Hunan University of Chinese Medicine; Hengyang Hospital affiliated to Hunan University of Chinese Medicine; Hubei Provincial Hospital of TCM; Jiangsu Province Hospital of TCM;, Shanxi Province Hospital of TCM; and Shanxi Hospital of Integrated Traditional and Western Medicine. Participants will be randomly assigned (on a 1:1 ratio) to the EA group or the sham EA group by a central randomization system performed by the Clinical Evaluation Center of the CACMS in Beijing. The randomization sequence will be generated by the ‘proc plan’ procedure of the SAS9.3 analytic software according to center-stratified, block randomization with a block size of six. Random number and group assignment will be offered by telephone, mobile phone, or website from the Clinical Evaluation Center of the CACMS. In this study, participants, outcome assessors, and statisticians will be blinded to treatment allocation. The flowchart and study design schedule are presented in Figure 1 and Table 1, respectively.

**Table 1:** Study design schedule

**Period**	**Screening**	**Baseline**	**Treatment(w1-6)**				**Follow**-**up(w7-30)**							
**Week (W)**	***W-1***	***W0***	***W2***	***W3***	***W4***	***W6***	***W15***	***W16***	***W17***	***W18***	***W27***	***W28***	***W29***	***W30***
Eligibility	X													
Informed consent	X													
Demography and medical history	X													
Physical examination	X													
Laboratory test	X													
1-h pad test		X	X			X								
72-h bladder diary		X	X		X	X	X	X	X	X	X	X	X	X
Weekly consumption of pads		X	X		X	X	X	X	X	X	X	X	X	X
Application of other treatments for SUI		X	X		X	X	X	X	X	X	X	X	X	X
ICIQ-UI Short form		X				X				X				X
Self-report assessment of therapeutic effect			X		X	X				X				X
Safety of EA			X		X	X								
Discomfort and acceptance of EA			X			X								
Assessment of blind method				X		X								
Adverse event			X			X	X	X	X	X	X	X	X	X
Compliance			X			X	X	X	X	X	X	X	X	X

### Ethics

The study is in accordance with the principles of the Declaration of Helsinki, and has been approved by the ethics committee boards of the participating hospitals (Ethics approval numbers: 2012EC007; 2013XL001-2; ECPJ-BDY-2013-04; 2013–033; 2013–7; TYLL2013; HN-LL-KY-2013-001-01; 2013EC001; HBZY2013-C007-01; 2013NL-013-04; 2013–02; 2012EC007) (Additional file 1). Written informed consent will be obtained from each subject before the patients enter the trial.

### Participants

The trial plans to recruit 500 women with pure SUI using research nurses or students majoring in acupuncture to do the recruiting.

Inclusion criteria are: (1) aged 40–75 years; (2) involuntary leakage of urine on effort, exertion, sneezing or coughing that stops when the stress ends [13]; (3) visible involuntary leakage from the urethra synchronous with increased abdominal pressure, or a pad weight gain >1 g in a 1-h pad test [13]; (4) no symptoms of urinary frequency and urgency; and (5) voluntarily join the research and sign the informed consent.

Exclusion criteria are: (1) urge urinary incontinence, mixed urinary incontinence, or overflow urinary incontinence; (2) having ever undergone an operation for urinary incontinence or on the pelvic floor; (3) female genital prolapse greater than degree 2; (4) symptomatic urinary tract infection; (5) residual urinary volume (RUV) > 30 mL; (6) maximum flow rate (Qmax) ≤20 mL/s; (7) limited in walking, stairs climbing and running; (8) receiving specific treatment for SUI, or taking medicine that may affect bladder function; (9) serious cardiovascular, cerebral, liver, kidney, or psychiatric disease, diabetes, multiple system atrophy, injury of cauda equina, or myeleterosis; (10) pregnancy or lactation; and (11) having a cardiac pacemaker, a metal allergy, or a severe needle phobia.

### Interventions

Acupuncture will be performed by registered acupuncturists with over 2 years of experience.

#### Treatment group

Acupuncture points (bilateral Zhongliao BL33 and Huiyang BL35) are selected based on evaluation of acupuncture literature, our pilot study and consensus of 10 Chinese acupuncture experts (Additional file 2). Before needling, sterile adhesive pads will be pasted onto the selected acupuncture points (for both groups, to ensure subject blinding). The adhesive pads (10 mm in diameter, 5 mm thick) are made of a special sponge and have a sticky side (Suzhou Medical Appliance Factory, China) (Additional file 3). The needles (0.30 mm in diameter, 75 mm in length, Hwato Brand, Suzhou Medical Appliance Factory, China) will be inserted at bilateral BL33 to a depth of 50–60 mm with an angle of 30-45° inward and downward. Bilateral BL35 are needled to a depth of 50–60 mm outward and upward slightly. Deqi, the needling sensations felt by a patient that indicates that the point has been stimulated, will be achieved by lifting, thrusting, and twirling the four needles evenly three times. The electric stimulators (SDZ-V electroacupuncture apparatus, Suzhou Medical Appliance Factory, China) will connect the bilateral BL33 points and the bilateral BL35 points and a continuous wave of 50 Hz frequency and an intensity of 1–5 mA will be applied. The intervention of treatment group is shown in Additional file 4.

#### Control group (sham EA)

Sham points, which are 1 cun lateral to BL33 and BL35, will be used to match each real point. Sham BL33 and BL35 will be needled through adhesive pads to the skin (for the fixation of needles) without penetrating the skin using blunt needles 0.30 mm in diameter, 25 mm in length (Hwato Brand, Suzhou Medical Appliance Factory, China). The needles will then be lifted, thrust, and twirled evenly three times to match the procedure undertaken for the treatment group. The sham electrode lines (Additional file 5), which look identical to the real ones but with the inner metal wire cut off, will be applied to bilateral sham BL33 and bilateral sham BL35, respectively, and a continuous wave of 50 Hz frequency and an intensity of 5 mA will be applied. When turned on, electric acupuncture apparatus looks normal but has no current output. The intervention of the control group is shown in Additional file 6.

For both groups, the needles will be retained for 30 min for each treatment session. The participants will be treated with EA three times a week, on alternate days, for six successive weeks; 18 sessions for each patient in total.

### Permitted and prohibited concomitant treatments

Throughout the trial, participants will be discouraged from undertaking any additional specific treatments for SUI including: medications such as duloxetine and imipramine; PFMT; feedback therapy; electrical or magnetic stimulation via pelvic floor, vagina or anus; and transcutaneous electrical nerve stimulation to the pelvic floor. For any unallowed treatment that has already been used, relevant information should be recorded in the patient’s case report form.

### Outcome measures

The primary outcome will be the change in the amount of urine leakage at week 6 from the baseline, measured by a 1-h pad test. The change at week 2 from the baseline will also be assessed. The 1-h pad test will be conducted based on the International Continence Society (ICS) guidelines [14] at the baseline, at week 2, and at week 6 (see Table 2). Secondary outcomes include seven items, the details of which are listed in Table 2.

**Table 2:** Trial outcomes

**Primary outcome**	**Time point**
Change in amount of urine leakage at week 6 from the baseline measured by 1-h pad test	Evaluated at week 6
**Secondary outcomes**	
1. 72-h IEF	Evaluated at weeks 6, 18, and 30. The change of average 72-h IEF from the baseline will be analyzed
	Based on a 72-h bladder diary, the average 72-h IEF of week 6 is calculated by averaging the IEF of weeks 2, 4, and 6; values of week 18 and 30 are calculated by averaging IEF of weeks 15–18 and 27–30, respectively
2. ICIQ-UI Short Form score	Evaluated at weeks 6, 18, and 30. The change of score from the baseline will be analyzed
3. Severity of urinary incontinence	Evaluated at weeks 6, 18, and 30. Change in the number and percentage of severity rating from the baseline will be analyzed
	The most severe degree during weeks 2, 4, and 6 will be taken as severity of urinary incontinence of week 6
	The most severe degree during weeks 15–18 and weeks 27–30 will be taken as severity of urinary incontinence of week 18 and week 30, respectively
4. Self-report assessment of therapeutic effect	Evaluated at weeks 6, 18, and 30
	A 4-point scale is used: no help, little help, medium help, and great help
5. Weekly consumption of pads	Evaluated at weeks 6, 18, and 30. The change in average consumption of pads from the baseline will be analyzed
	The pads consumption of weeks 6, 18, and 30 will be calculated by averaging the weekly usage of pads during weeks 1–6, weeks 7–18, and weeks 19–30, respectively
6. Application of other treatment for SUI (including drugs and other specialist treatment)	Evaluated at weeks 6, 18, and 30. The number of patients who used other treatments for SUI during the trial will be analyzed between the two groups
7. Subgroup analysis stratified by incontinence severity	Evaluated at weeks 6, 18, and 30
	Stratified by incontinence severity at the baseline, the correlation between the rating of incontinence severity and the treatment effect will be analyzed. Different outcomes of treatment effect are used in each evaluation: week 6, the change in amount of urine leakage at week 6 from the baseline measured by 1-h pad test; week 18, the change of average 72-h IEF at week 18 from the baseline; and week 30, the change of average 72-h IEF at week 30 from the baseline

### Assessment of EA safety

Acupuncture is considered to be a generally safe procedure [15]; however, all adverse events will be recorded in detail. In this study, EA-related adverse events would mainly refer to broken needles, fainting due to the needling procedure, continuous post-needling pain lasting >2 h, local infection, hematoma, bleeding, and other events that can be caused by acupuncture (such as fatigue, headache, insomnia, and dizziness).

### Assessment of the subject blinding success rate

**Figure 2:**
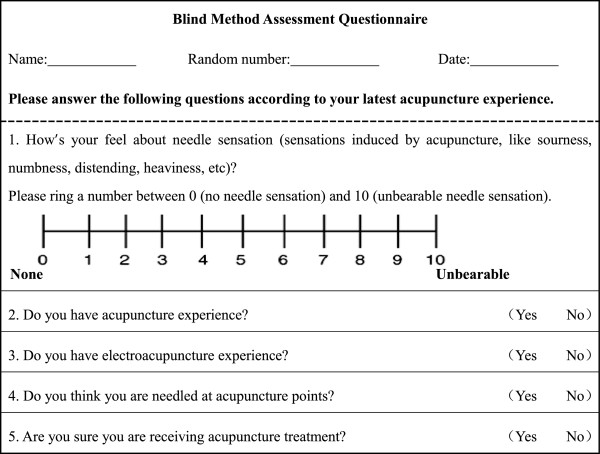
Blind method assessment questionnaire.

Two of the study centers will be randomly selected to measure the success rate of subject blinding via analyzing questionnaire response (Figure 2). The questionnaire will be completed by subjects within 5 min after any treatment session in weeks 3 and 6. The percentage of subjects from each group who believe that they received a true EA treatment (regardless of whether they received an actual true or actual sham treatment) will be recorded as P1 in week 3 and P2 in week 6. The subject blinding success rate will be defined as the average of P1 and P2. The difference in the subject blinding success rates between the two groups will be analyzed.

### Statistical methods

#### Sample size calculation

We conducted a pilot study comparing the effects of deep EA with shallow EA on women with SUI in Guang’an men Hospital of CACMS between June 2011 and February 2012. Results from our pilot study revealed that: compared with the baseline, the volume of urine leakage from the 1-h pad test decreased by 2.10 g, with a standard deviation of 2.26, after 4 weeks deep EA treatment, and by 0.64 g with a standard deviation of 0.97 after 4 weeks shallow EA treatment [16]. We performed sample size calculation according to results of our pilot study because no placebo control trial has been reported. A sample size of 117 in each group will be sufficient to detect a clinically important difference of 1 g on the volume of urine leakage from a 1-h pad test, assuming a standard deviation of 2.61 (considering a larger variability in multicenter trial, the pooled standard deviation of pilot study 1.74 was expanded by 1.5 times), using a one-tailed *t*-test of the difference between means, a significance level of 5%, and a power of 90%. The calculation is based on the assumption that measurements on volume of urine leakage from a 1-h pad test are normally distributed. This number has been increased to 140 per group (total of 280), to allow for a predicted drop-out rate of 20%. As our pilot study had a small sample size (*n*=40) and as we will undertake subgroup analysis, we expanded the sample size to 500 cases (250 cases in each group) to enhance the reliability of the study.

#### Statistical analysis

Statistical analysis will be performed by the Clinical Evaluation Center of CACMS in Beijing and the Department of Biostatistics, University of Washington, Seattle, United States. The statistician will be blinded from the allocation of groups. PASW Statistics 20.0 and SAS9.0 statistical software will be used for data analysis. The level of significance is established at α<0.05 with a two-tailed test.

The main objective is to compare the change in the amount of urine leakage at week 6 from the baseline between the treatment group and the control group. The null hypothesis is that the treatment group has the same change as the control group, while the alternative hypothesis is that the treatment group shows a greater decrease.

Continuous data will be represented by the average, standard deviation, median, minimum value, and maximum value, whereas categorical data will be represented by percentages. For comparison with the baseline, a *t*-test or non-parametric test will be used for continuous data, and non-parametric tests for categorical data. For comparison of two independent samples: if the residual are normally distributed, the analysis of covariance (ANCOVA) will be used for the primary outcome and subgroup analysis stratified by incontinence severity, a *t*-test for other continuous data, and a chi-square test for categorical data; if the residual are abnormally distributed, a non-parametric test will be used for both continuous and categorical date. Detailed contents and methods are as follows.

1. Case distribution and compliance analysis: The case distribution of both groups in every center will be described. The total drop-out rate and the drop-out rate due to adverse events, of both groups, will be compared using Fisher’s exact test.

2. Analysis of baseline characteristics.

3. Analysis of compliance: The implementation of protocol intervention will be compared between the two groups. Compliance will be evaluated according to the case report forms.

4. Analysis of efficiency: Intention-to-treat (ITT) analysis will be used to assess the validity of the study. Efficiency analysis will be based on the ITT population which is defined as all randomized participants. Missing data and non-compliance data will be included in the efficiency analysis. Missing data, from subjects without any treatment after randomization, or without any valid data of evaluation although treated, will be analyzed by multiple imputation by the inverse probability weighting method. Non-compliance data will be analyzed by the instrumental variable.

5. Analysis of primary outcome: In this study, we will use the change in the amount of urine leakage at week 6 from the baseline as the primary outcome. To adjust for baseline imbalance, a comparison of the decreased value of urine leakage between the groups will be done by ANCOVA using baseline and centers as covariates, when the residual are normally distributed. Meanwhile, a covariance model including interactions between centers and group will be made to analyze the center effect. Causes will be further analyzed if any center effect is identified. Comparison of decreased value of urine leakage between groups will be analyzed by a non-parametric test if the residual are abnormally distributed with neglect of baseline balance.

6. Analysis of secondary outcomes:

A 72-h incontinence episode frequency (IEF), the International Consultation on Incontinence Questionnaire-Urinary Incontinence (ICIQ-UI) Short Form score, and weekly consumption of pads of weeks 6, 18, and 30 will be compared with their respective baselines, and a *t*-test or non-parametric test will be used for comparison among groups.

For severity of urinary incontinence based on a 72-h bladder diary and a self-report assessment of therapeutic effect, the case number and percentage of each rank of weeks 6, 18, and 30 will be compared with their baselines, and a chi-square test or non-parametric test will be used for comparison among groups.

Although we will exclude patients already receiving some form of treatment for SUI at the trial outset and will discourage participants from taking additional treatments during the trial period, we anticipate that some patients will not adhere to this. In these instances the patients will record the treatments received and/or medications taken and the number of patients and treatment sessions of the treatment period and two follow-up periods will be described, and chi-square tests or non-parametric tests will be used for comparison among groups.

To assess the effect of EA for different degrees of SUI, subgroup analysis will be stratified by incontinence severity measured by a 1-h pad test at the baseline. In comparison among the groups, changes in the amount of urine leakage at week 6 from the baseline will be analyzed by ANCOVA or nonparametric tests. Changes in the 72-h IEF at weeks 18 and 30 will be analyzed by covariance analysis, non-parametric tests, or longitudinal data model.

7. Analysis of safety: Adverse events will be listed and analyzed using a chi-square test or Fisher’s exact test. Severe adverse events should be listed in detail. EA-related adverse events are to be listed in detail and compared among groups.

8. Analysis of blind method: The number and percentage of subjects choosing true EA in the selected two centers will be established. A chi-square test or non-parametric test will be used for comparison among groups.

## Discussion

This trial is expected to provide convincing evidence that EA has an efficacy effect, and not a placebo effect, for pure SUI. Extant literature shows that acupuncture is probably effective for SUI; however, more evidence is needed [10,17,18]. In existing studies, there are no examples of placebo being used as a control. To our knowledge, this is the first acupuncture trial applying sham EA as a control in studying SUI. To maximally exclude the placebo effect, rigorous methodological designs are followed. We will use non-acupoints, non-penetrative needling, and special electrode lines with no current output for control. Points used in this trial are located on the lumbosacral region; hence, participants are unable to see their treatment. In addition, participants will receive treatments alone at different times to avoid communication with each other. By following these methods, participants can be blinded successfully, and the efficacy of EA could be confirmed if the results of the treatment group prove superior to the control group.

To evaluate the effect of SUI, a pad test, a bladder diary and the ICIQ-SF are recommended by the ICS. Among various pad test types, the 1-h pad test and the 24-h pad test [19] are most commonly used. These two pad tests were considered to be of good accordance with a subjective assessment of SUI [20] or ICIQ-SF [21]; the 1-h pad test was adopted in this trial to maintain patient compliance.

It is worth mentioning that the needling methods of BL33 and BL35 to be used in our study are different from the routine needling of these points. In routine needling, both BL33 and BL35 should be needled perpendicularly to a depth of 0.8-1 cun, which is no more than 25 mm. In our study, an oblique needling with a depth of 50–60 mm is needed, since we hold that neural regulation is the reason that acupuncture takes effect for SUI. Deep needling of BL33 and BL35 can directly stimulate the pudendal nerve and the anterior branches of the third sacral nerve, and thus act to strengthen contraction of the pelvic floor muscle and the striated urethral sphincter.

Overall, acupuncture is a comparatively safe therapy [22] and is gradually gaining acceptance in urology as an effective treatment for erectile problems, lower urinary tract symptoms and chronic prostatitis [23]. Since deep needling and strong stimulation are used in the treatment group, the evaluation of EA safety is necessary.

In conclusion, results of this trial are expected to confirm whether EA is effective for women with pure SUI, and whether this effectiveness is an acupuncture efficacy rather than a placebo effect.

## Trial status

This trial is currently recruiting participants, and will be completed by 31 December 2014.

## Abbreviations

EA: Electroacupuncture; ITT: Intent to treat; LUTS: Lower urinary tract symptoms; PFMT: Pelvic floor muscle training; RCTs: Randomized controlled trials; SUI: Stress urinary incontinence; VAS: Visual analogue scale.

## Competing interests

The authors declare that they have no competing interests.

## Authors’ contributions

ZSL and BYLwere responsible for the design, supervision of the study, and revision of the manuscript. HFX drafted the manuscript. JL did the trial registration and manuscript revision. SYY designed statistical plan. YLC and LYH participated in the revision of the manuscript and coordination of the study. JNW and RSD participated in data acquisition. All authors read and approved the final manuscript.

## Supplementary Material

Additional file 1 Ethical approval of all participating hospitals. Click here for file

Additional file 2 Acupuncture points. Click here for file

Additional file 3 Adhesive pad. Click here for file

Additional file 4 Treatment group. Click here for file

Additional file 5 Sham electrode line. Click here for file

Additional file 6 Control group. Click here for file
