# Supplementary figures and images for: The efficacy and safety of electroacupuncture for women with pure stress urinary incontinence: study protocol for a multicenter randomized controlled trial
Source: Trials. 2013 Sep 30;14:315. doi: 10.1186/1745-6215-14-315 (PMC3850726; doi:10.1186/1745-6215-14-315)

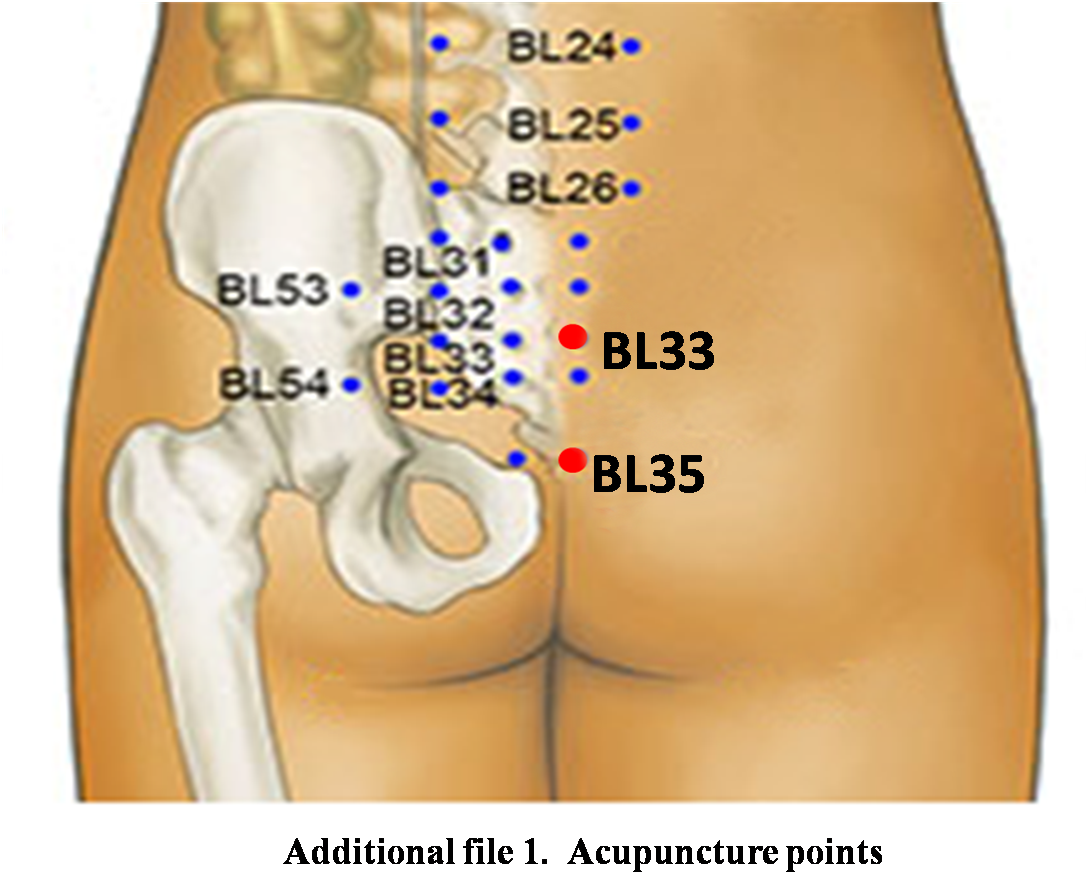

Supplement: Additional file 1 — Ethical approval of all participating hospitals. [file 1745-6215-14-315-S1.png]

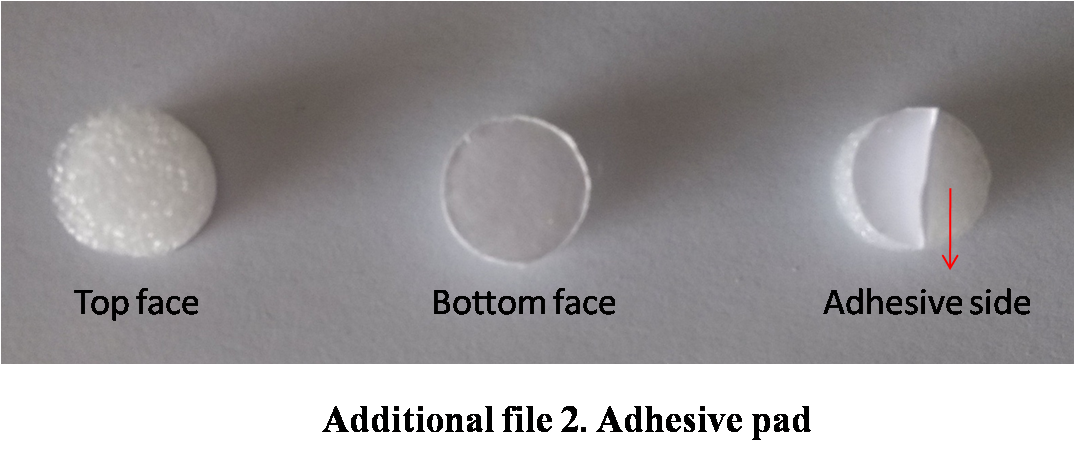

Supplement: Additional file 2 — Acupuncture points. [file 1745-6215-14-315-S2.png]

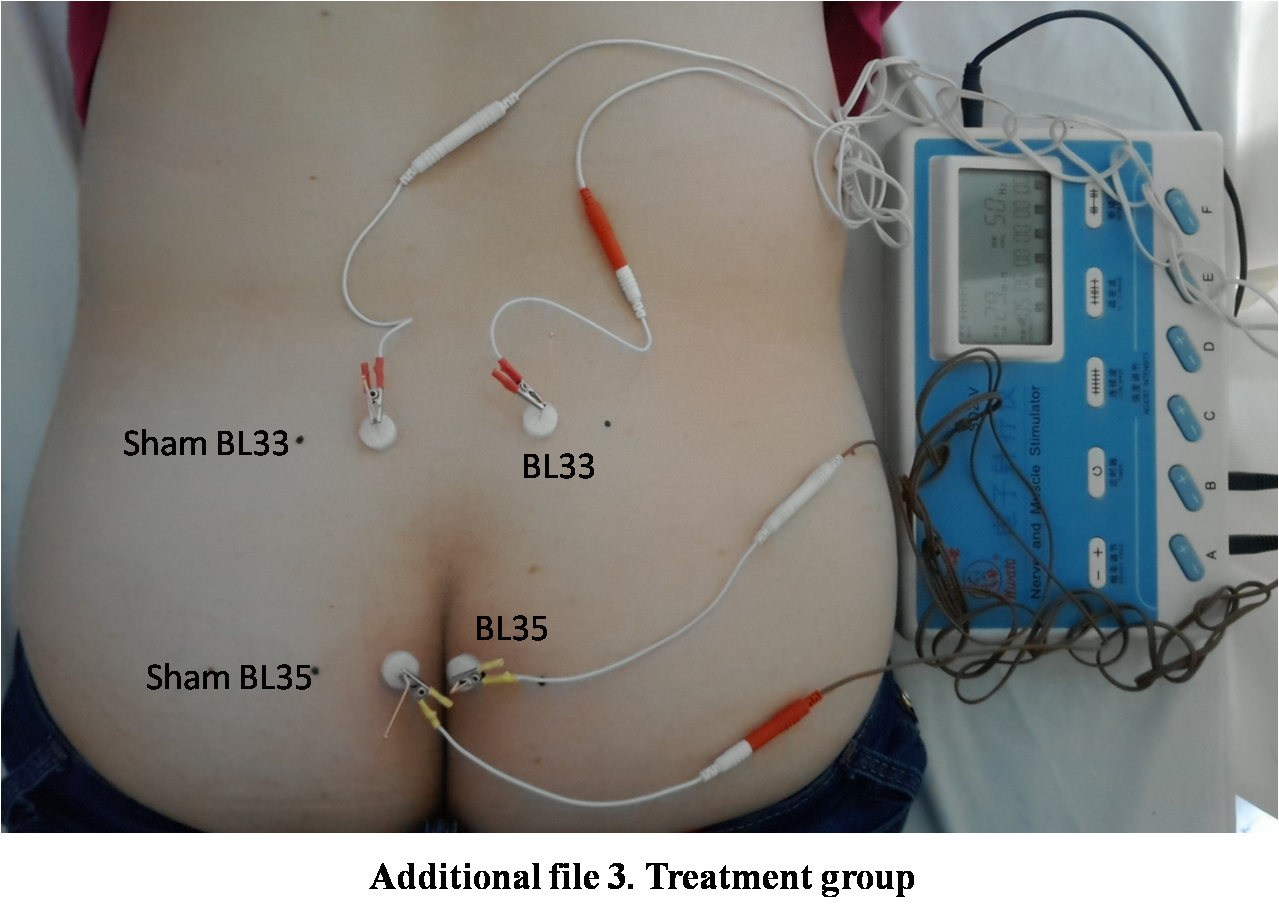

Supplement: Additional file 3 — Adhesive pad. [file 1745-6215-14-315-S3.png]

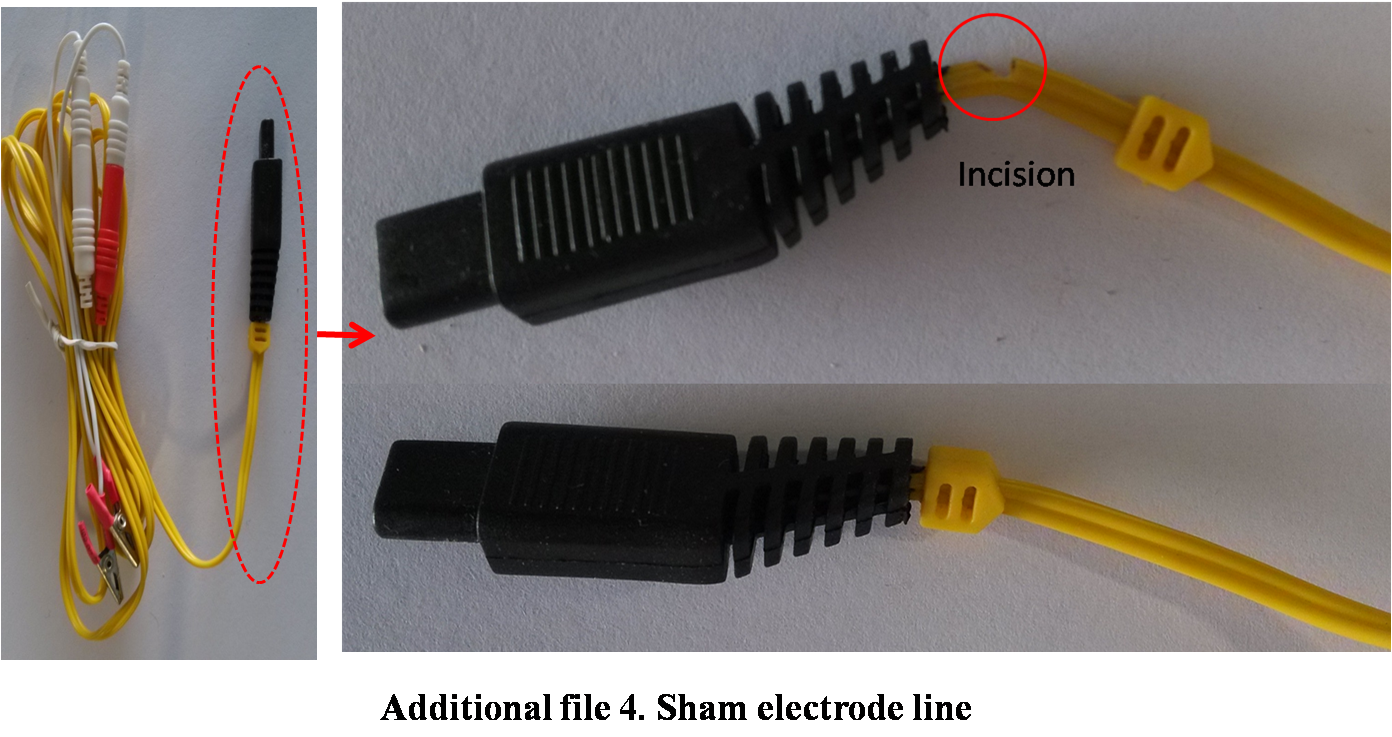

Supplement: Additional file 4 — Treatment group. [file 1745-6215-14-315-S4.png]

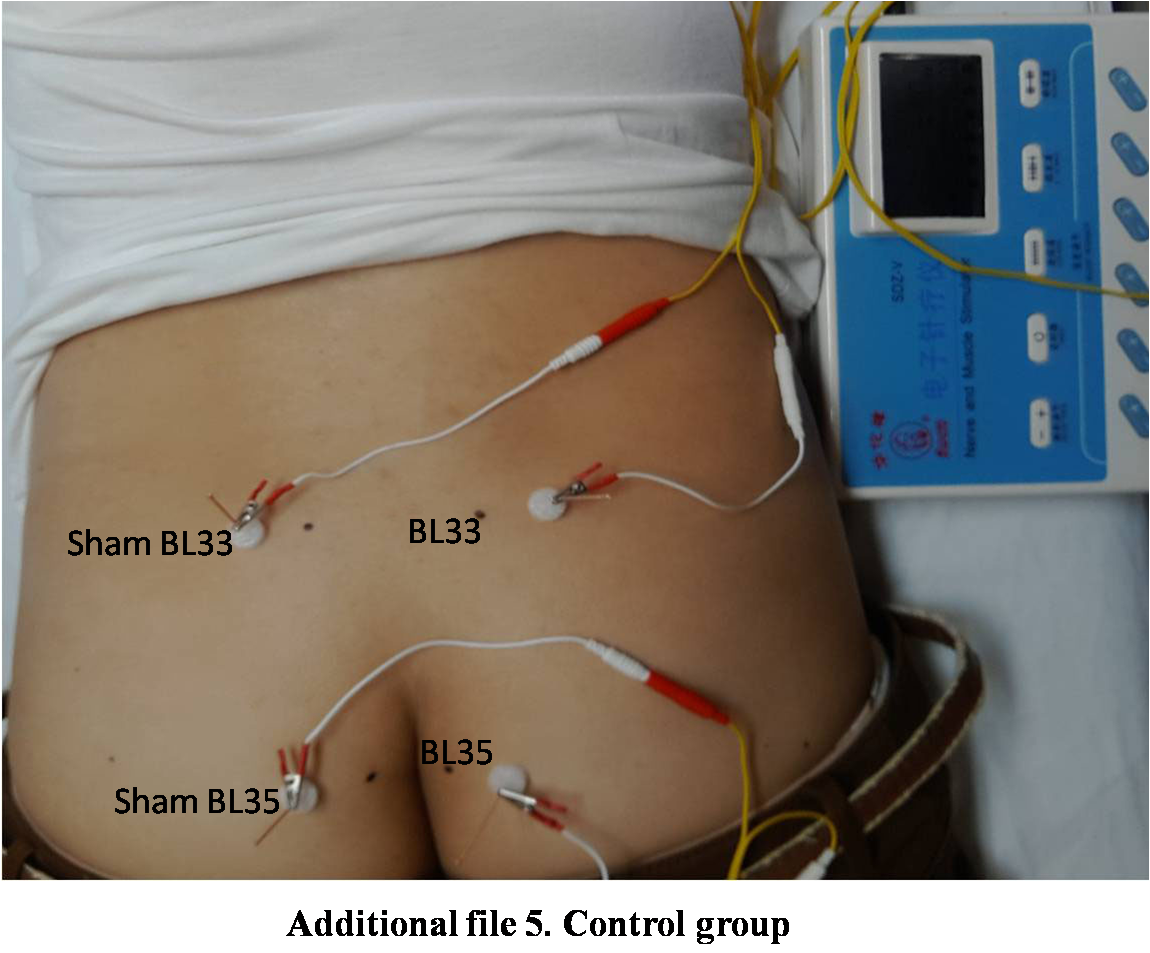

Supplement: Additional file 5 — Sham electrode line. [file 1745-6215-14-315-S5.png]
