# Supplementary material for: The efficacy and safety of electroacupuncture for women with pure stress urinary incontinence: study protocol for a multicenter randomized controlled trial
Source: Trials. 2013 Sep 30;14:315. doi: 10.1186/1745-6215-14-315 (PMC3850726; doi:10.1186/1745-6215-14-315)
Supplement: Additional file 6 — Control group. [file 1745-6215-14-315-S6.pdf]

# Ethical approvals of all participating hospitals

This trial is to be conducted in 12 hospitals. The ethical review was firstly submitted to IRB of the principle organization, Guang'an men Hospital, and then to IRB of other participating hospitals. This trial has gained approval from all of the IRBs.

Ethical approvals are attached in the following sequence.

1. Guang'an men Hospital, China Academy of Chinese Medical Sciences
2. Xiyuan Hospital of China Academy of Chinese Medical Sciences
3. Dongzhimen Hospital affiliated to Beijing University of Chinese Medicine
4. Yueyang Hospital of Integrated Traditional Chinese and Western Medicine affiliated to Shanghai University of Traditional Chinese Medicine
5. West China Hospital of Sichuan University
6. First Teaching Hospital of Tianjin University of TCM
7. First Hospital of Hunan University of Chinese Medicine
8. Hengyang Hospital affiliated to Hunan University of Chinese Medicine
9. Hubei Provincial Hospital of TCM
10. Jiangsu Province Hospital of TCM
11. Shanxi Province Hospital of TCM
12. Shanxi Hospital of Integrated Traditional and Western Medicine

**Attachment 1: Ethical approval of Guang'an men Hospital, China Academy of Chinese Medical Sciences**



# 伦理审查批件

| | | | |
|---|---|---|---|
| 项目名称 | 电针治疗女性单纯性压力性尿失禁有效性和安全性多中心随机对照试验 | | |
| 项目编号 | 2012EC007 | 项目来源 | “十二五”国家科技支撑计划 |
| 牵头单位 | 中国中医科学院广安门医院 | | |
| 申办者（如有） | / | | |
| 主要研究者 | 刘志顺 | | |
| 审查类别 | 初始审查 | 审查方式 | 会议审查 |
| 审查日期 | 2012.12.14 | 审查地点 | 广安门医院行政楼四楼会议室 |
| 审查委员 | 殷海波，朴炳奎，林兰，谢利民，曹炜，冯玲，赵军，胡镜清，吴萍，顾丽贞，沈瑞英 | | |
| 批准文件 | 研究方案（VERSION 1.0_20121105），知情同意书（V1.0） | | |
| 审查意见 | 根据卫生部《涉及人的生物医学研究伦理审查办法（试行）》、国家药品食品监督管理局《药物临床试验质量管理规范》、《医疗器械临床试验规定》、《药物临床试验伦理审查工作指导原则》国家中医药管理局《中医药临床研究伦理审查管理规范》以及《赫尔辛基宣言》和国际医学科学组织委员会颁布的《人体生物医学研究国际道德指南》的伦理原则，经本伦理委员会审查，同意按所批准的临床研究方案、知情同意书、招募材料开展本项研究。<br>请遵循 GCP 原则、遵循伦理委员会批准的方案开展临床研究，保护受试者的健康与权利。<br>研究过程中若变更主要研究者，对临床研究方案、知情同意书、招募材料等的任何修改，请申请人提交修正案审查申请。<br>如发生严重不良事件以及影响研究风险受益比的非预期不良事件，请申请人及时提交严重不良事件报告。<br>请按照伦理委员会规定的年度/定期跟踪审查频率，申请人在截止日期前1个月提交研究进展报告；申办者应当向组长单位伦理委员会提交各中心研究进展的汇总报告；当出现任何可能显著影响试验进行或增加受试者危险的情况时，请申请人及时向伦理委员会提交书面报告。<br>研究纳入了不符合纳入标准或符合排除标准的受试者，符合中止试验规定而未让受试者退出研究，给予错误治疗或剂量，给予方案禁止的合并用药等没有遵从方案开展研究的情况；或可能对受试者的权益/健康以及研究的科学性造成不良影响等违背 GCP 原则的情况，请申办者/监查员/研究者提交违背方案报告。<br>提前终止或完成临床研究，请及时提交结题报告。 | | |
| 有效期 | 2012 年 12 月 20 日～2013 年 12 月 19 日 | | |
| 联系人与联系电话 | 乔洁 010-88001552 | | |
| 主任委员签字 | [signature] | | |
| | 中国中医科学院广安门医院伦理委员会（盖章） | | |
| | 日期：2012 年 12 月 21 日 | | |

中国中医科学院广安门医院伦理委员会

**Attachment 2: Ethical approval of Xiyuan Hospital of China Academy of Chinese Medical Sciences**

# 中国中医科学院西苑医院医学伦理委员会审查批件

批件号：中国中医科学院西苑医院医学伦理委员会 **2013XL001-2**

| | |
|---|---|
| 审查日期 | 2013 年 1 月 25 日 |
| 审查地点 | 北京海淀区西苑操场 1 号中国中医科学院西苑医院医学伦理委员会 |
| 课题编号 | 2012BAI24B01 |
| 项目名称 | 电针治疗女性单纯性压力性尿失禁有效性和安全性 ——多中心随机对照试验 |
| 审查文件 | 伦理审查申请书、研究者手册、研究者履历、CRF、患者日记等；<br>研究方案：版本号：VERSION 1.0_20121106，版本日期：2012 年11 月6 日<br>修正的知情同意书:版本号:VERSION 1.0 20120220，版本日期：2013 年2 月20 日 |
| 课题组织单位 | 国家科技部“十二五”国家科技部 |
| 临床研究单位 | 中国中医科学院广安门医院、北京中医药大学东直门医院、 四川大学华西医院、中国中医科学院西苑医院、湖南中医药大学附属衡阳医院、湖南省中医院、上海中医药大学附属岳阳中西医结合医院、天津中医药大学第一附属医院、陕西省中医医院、江苏省中医院、山西中医学院中西医结合医院、湖北省中医院 |
| 主要研究者 | 陆永辉（副主任医师） -本中心 |
| 会议审查委员 | 曹云、衷敬柏、尚晓泓、尹秀云、张广生、杨志旭、于振宣、韩梅、李涛、房定亚、闫小平 |
| 审查意见 | 根据中华人民共和国国家药品食品监督管理局 2003 年颁布实施的《药物临床试验质量管理规范》、2010 年 11 月颁布的《药物临床试验伦理审查工作指导原则》以及《赫尔辛基宣言》的伦理原则，经本伦理委员会会议审查，审查结果为“作必要的修正后同意”，具体意见如下：<br>（1）主要研究者符合国家相关规定；<br>（2）研究方案的设计基本符合科学性、伦理性原则。临床筛选患者做残余尿检查时不应让患者大量饮水。<br>（3）修正后的知情同意书语言通俗易懂，信息充分。<br>（4）给予伦理审查批件。如临床试验方案有任何修改，主要研究者更换等，需重新审查，获得批准后执行。<br>暂停/提前终止/完成临床研究，请及时通知伦理委员会。<br>**本批件有效期一年，请于 2014 年 2 月 21 日前 1 个月提交跟踪审查申请报告。**<br>发现影响受试者参加研究意愿的违反方案情况应及时报告 |
| 联系电话 | 伦理委员会秘书 訾明杰（010）62835646 |
| 主任委员 | 曹云 |
| 盖　　章 | 中国中医科学院西苑医院医学伦理委员会 |
| 日　　期 | 2013 年 2 月 22 日 |

**Attachment 3: Ethical approval of Xiyuan Hospital of China Academy of Chinese Medical Sciences**

北京中医药大学东直门医院医学伦理委员会
IRB of Dongzhimen Hospital affiliated to Beijing University of Chinese Medicine

# 伦理审查批件

Approval Notice Template

受理序号：ECSL-BDY-2013-04　　　　批件号：ECPJ-BDY-2013-04

项目名称：电针治疗女性单纯性压力性尿失禁有效性和安全性多中心随机对照试验

申办单位：东直门医院　　　　主要研究者：赵吉平

项目类别：国家科技支撑项目　　　　批文号/课题编号：2012BAI24B01

方案版本号：1.0_20121105　　　　方案批准日期：2012.11.5

知情同意书版本号：V1.0　　　　知情同意书批准日期：2012.11.5

伦理审查方式：☑会议审查　　☐快速审查

应到会 15 人，出席本次会议人员 9 人，回避 0 人，缺席 6 人

| | |
|---|---|
| 根据中华人民共和国国家食品药品监督管理局（SFDA）《药物临床试验伦理审查工作指导原则》（2010 年）、《药物临床试验质量管理规范》（2003）、《中药品种保护指导原则》（2009），世界医学会《赫尔辛基宣言》（2008），卫生部《涉及人的生物医学研究伦理审查办法》（2007），国家中医药管理局《中医药临床研究伦理审查管理规范》（2010）以及国际医学科学组织委员会《人体生物医学研究国际道德指南》（2002）的伦理原则，经本伦理委员会审查决定： | ☑同意临床研究方案<br>☐不同意临床研究方案<br>☐终止临床研究方案<br>☐暂停临床研究方案 |

审查意见：

同意临床研究

注：本批件自签发日期有效期一年，研究负责人必须严格使用经审查同意的知情同意书文本和研究方案。如伦理审查批件失效时不能完成所有的临床研究（包括统计分析），请在本批件失效前一个月，递交持续审查申请。如研究结束并在审查有效期内，请递交研究结题报告。研究中发生涉及受试者或其他人风险的任何预期或非预期的不良事件，应立刻报告本伦理委员会；任何研究方案、知情同意书的修改包括研究人员得变更，必须递交研究方案修改申请表，经伦理委员会审查获得批准后执行。

主任委员☑副主任委员☐签字：[illegible]　　时 间：2013年 2月 5 日

北京中医药大学东直门医院医学伦理委员会　　地 点：第一会议室

本项目持续审查频率☐3个月 ☐6个月 ☑12个月　联系人：商建伟 （010）84013229

北京中医药大学东直门医院医学伦理委员会

IRB of Dongzhimen Hospital affiliated to Beijing University of Chinese Medicine

# 会议签到表

Meeting attendance sheet

| | | | |
|---|---|---|---|
| **项目名称** | 电针治疗女性单纯性压力性尿失禁有效性和安全性多中心随机对照试验 | | |
| **会议时间** | 2 月 1 日 | **会议地点** | 第一会议室 |

| 成员 | 性别 | 伦理委员会职务 | 专业 | 签名 |
|---|---|---|---|---|
| 李澎涛 | 男 | 主任 | 脑病 | |
| 高　颖 | 女 | 副主任 | 脑病 | [illegible] |
| 叶永安 | 男 | 副主任 | 消化 | |
| 柳红芳 | 女 | 副主任 | 肾病内分泌 | [illegible] |
| 张永涛 | 男 | 委员 | 呼吸 | [illegible] |
| 王新月 | 女 | 委员 | 消化 | |
| 杨博华 | 男 | 委员 | 周围血管 | |
| 鲁卫星 | 男 | 委员 | 心血管 | [illegible] |
| 王蓬文 | 女 | 委员 | 药理学 | [illegible] |
| 曹俊岭 | 男 | 委员 | 药剂学 | [illegible] |
| 刘　凯 | 男 | 委员 | 法律代表 | |
| 贺海东 | 男 | 委员 | 医疗器械 | [illegible] |
| 张胜利 | 男 | 委员 | 群众代表 | [illegible] |
| 陈信义 | 男 | 委员 | 血液肿瘤 | |
| 彭淑莲 | 女 | 委员 | 乳腺外科 | [illegible] |

**Attachment 4: Ethical approval of Yueyang Hospital of Integrated Traditional Chinese and Western Medicine affiliated to Shanghai University of Traditional Chinese Medicine**

上海中医药大学附属岳阳中西医结合医院伦理委员会

IRB of Yueyang Hospital of Integrated Traditional Chinese and Western Medicine, Shanghai University of TCM

**伦理审查批件**

Approval Notice Template

伦理审议批件号：上海中医药大学附属岳阳中西医结合医院伦理委员会 2013 伦理审查 033 号 (2013-033)

| | | | |
|---|---|---|---|
| 研究名称 | 电针治疗女性单纯性压力性尿失禁有效性和安全性多中心随机对照试验 | | |
| 研究类型 | 临床试验 | 研究周期 | 两年 |
| 研究单位和研究者 | 上海中医药大学附属岳阳中西医结合医院　陈跃来 | | |
| 伦理委员会审议成员 | 金利国、王雪文、徐玲玲、史晓、常时新、陈云飞、樊民胜、周正、任力 | | |
| 伦理委员会地址 | 上海市虹口区甘河路 110 号 | | |
| 审议时间 | 2013 年 4 月 27 日 | | |
| 审议结论 | 根据中华人民共和国国家药品监督管理局 2003 年颁布实施的《药物临床试验质量管理规范》以及《赫尔辛基宣言》和国际医学科学组织委员会颁布的《人体生物医学研究国际道德指南》的道德原则。本伦理委员会的全体成员审阅并讨论了下列有关材料：<br>1、临床课题伦理审查申请表<br>2、研究方案（1.0 2012.11.06）<br>3、知情同意书（2.0 2013.05.10）<br>4、主要研究者简历<br>5、招募广告<br>6、CRF 表（1.0 2012.11.09）<br>7、研究人员名单<br>8、其他资料：病例筛选表；尿垫使用情况记录表；排尿日记；<br><br>本伦理委员会经表决同意你们自即日起开展“电针治疗女性单纯性压力性尿失禁有效性和安全性多中心随机对照试验”；<br>并要求：上述资料未经本委员会批准，不得作任何修改；试验过程中如发生严重不良事件，应立即（24 小时内）报告本委员会；如临床试验方案、知情同意书及研究者有任何更改，应及时通知伦理委员会，得到重新批准。 | | |
| 主任委员签字 | [signature] | | |
| 备注 | 该批件有效期一年，自批件生效日起 12 月内未完成研究的，请向伦理委员会提交跟踪审查申请。<br>联系人：肖夏懿　电话：65161782*2419 | | |

上海中医药大学附属岳阳中西医结合医院

医学伦理委员会（盖章）

2013 年 5 月 20 日

## 上海中医药大学附属岳阳中西医结合医院伦理委员会

IRB of Yueyang Hospital of Integrated Traditional Chinese and Western Medicine, Shanghai University of TCM

### 会议签到表

Sign-in Sheet of Full Board Meeting

会议日期：2013 年 4 月 27 日

审查项目：电针治疗女性单纯性压力性尿失禁有效性和安全性多中心随机对照试验

伦理委员会到会委员签名：

| 姓名 | 性别 | 专业情况 | 签名 |
|---|---|---|---|
| 金利国 | 男 | 医学、管理 | [illegible] |
| 陈云飞 | 男 | 医学 | [illegible] |
| 常时新 | 男 | 医学 | [illegible] |
| 徐玲玲 | 女 | 药学 | [illegible] |
| 史　晓 | 女 | 中医学 | [illegible] |
| 王雪文 | 女 | 护理学 | [illegible] |
| 樊民胜 | 男 | 社会科学、伦理学 | [illegible] |
| 周　正 | 男 | 法律 | [illegible] |
| 任　力 | 男 | 法律 | [illegible] |

**Attachment 5: Ethical approval of West China Hospital of Sichuan University**

# 四川大学华西医院临床试验与生物医学伦理专委会审查批件

2013年 审（7）号

| | | | |
|---|---|---|---|
| 科室（专业）：中西医结合科 | | 项目负责人姓名及职称：李宁　副主任医师 | |
| 项目名称 | 电针治疗女性单纯性压力性尿失禁有效性和安全性—多中心随机对照试验 | | |
| 研究方案 | 版本号：无 | 版本日期：2013.1.7 | |
| 知情同意书 | 版本号：修订版 | 版本日期：2013.1.29 | |

审查意见：

1. 研究者资质符合伦理要求。
2. 研究方案及知情同意书基本符合伦理要求。

审查结果：■同意　□作必要修正后同意　□修正后再审　□不同意　□终止或暂停

请遵循我国相关法律、法规和规章（SFDA《药物临床试验质量管理规范》（2003）、《医疗器械临床试验规定》（2004）、WMA《赫尔辛基宣言》和CIOMS《人体生物医学研究国际道德指南》、卫生部《涉及人的生物医学研究伦理审查办法（试行）（2007）》），遵循伦理委员会批准的方案和知情同意书开展临床试验(研究)，保护受试者的健康与权利。

在试验（研究）过程中，若变更主要研究者，对临床研究方案、知情同意书等的任何修改，请申请人提交修正案审查申请。

发生严重不良事件，请申请人及时提交严重不良事件报告；紧急报告之后，尽快提交详细的严重不良事件随访报告。

请递交年度和定期跟踪审查报告；当出现任何可能显著影响试验（研究）进行或增加受试者危险的情况时，请申请人及时向伦理专委会提交书面报告。

试验（研究）纳入了不符合纳入标准或符合排除标准的受试者，符合中止试验（研究）规定而未让受试者退出试验（研究），给予错误治疗或剂量，给予方案禁止的合并用药等没有遵从方案开展研究的情况；或可能对受试者的权益/健康、以及研究的科学性造成不良影响等违背伦理原则与规范的情况，请申办者/监查员/研究者提交违背方案报告。

申请人暂停或提前终止临床试验（研究），请及时提交暂停/终止试验（研究）报告。完成临床试验（研究），请申请人提交结题报告。

单位（章）

主任委员（签名）：

2013 年 2 月 22日

**Attachment 6: Ethical approval of First Teaching Hospital of Tianjin University of TCM**



天津中医药大学第一附属医院医学伦理委员会
IEC of The First Affiliated Hospital of Tianjin University of Traditional Chinese Medicine

# 审 查 批 件

Approval Notice

伦理批件号：TYLL2013[E]字 001

根据卫生部《涉及人的生物医学研究伦理审查办法》(2007)、国家中医药管理局《中医药临床研究伦理审查管理规范》(2010)、国家食品药品监督管理局《药物临床试验伦理审查工作指导原则》(2010)、《药物临床试验质量管理规范》(2003)，以及世界医学会《赫尔辛基宣言》(2008)、国际医学科学组织理事会《人体生物医学研究国际伦理指南》(2002)的伦理原则，经天津中医药大学第一附属医院医学伦理委员会 2013 年 3 月 8 日快速审查，同意由申办者天津中医药大学第一附属医院和主要研究者傅立新共同申请的电针治疗女性单纯性压力性尿失禁有效性和安全性-多中心随机对照试验项目开展临床研究工作。

请申办者、研究人员严格遵循 GCP 规定和本伦理委员会批准的方案（版本号：VERSION1.0_20121106 版本日期：20121106）、知情同意书（版本号：VERSION1.0_20121106 版本日期：20121106）开展临床研究。在研究开始前，须完成临床试验注册。该项目进行中如发生下列情况，须及时书面报告本伦理委员会：①对临床方案、知情同意书等的任何修改；②更换主要研究者；③发生严重不良事件；④出现任何可能影响试验进行或增加受试者危险的情况；⑤出现违反方案情况；⑥暂停或提前终止临床研究。

本伦理委员会将对该项目跟踪审查。

请于 2014 年 3 月 8 日前 1 个月提交研究进展报告。

该项目完成后，请向本伦理委员会提交结题报告。

本批件有效期为 2013 年 3 月 8 日至 2016 年 3 月 8 日。

天津中医药大学第一附属医院医学伦理委员会

主任委员签字：张金锺

日　　期：2013.3.8

联系人：贾景蕴　　　　联系电话：022-27432276

**Attachment 7: Ethical approval of First Hospital of Hunan University of Chinese Medicine**

编号：AF/SC-08/01.0

# 伦理审查批件

| | | | |
|---|---|---|---|
| 批件号 | 湖南中医药大学第一附属医院伦理委员会 HN-LL-KY-2013-001-01 | | |
| 项目名称 | 电针治疗女性单纯性压力性尿失禁有效性和安全性-多中心随机对照试验 | | |
| 项目来源 | “十二五”国家科技支撑计划 2012BAI24B01 | | |
| 研究单位 | 中国中医科学院广安门医院、湖南中医药大学第一附属医院等 | | |
| 主要研究者 | 章薇 | | |
| 审查类别 | 初始审查 | 审查方式 | 会议审查 |
| 审查日期 | 2013.1.23 | 审查地点 | 医院会议室 |
| 审查委员 | 郭志华，贺菊乔，赵艳玲，陈其华，黄孟君，张志国，张月娟，谭　劲，谢海波，钟　晓，管小平 | | |
| 批准文件 | 临床研究方案（版本号:VERSION1.0-20121106）<br>知情同意书（版本号: VERSION1.0-201201109） | | |

审查意见

根据卫生部《涉及人的生物医学研究伦理审查办法（试行）》(2007)、SFDA《药物临床试验质量管理规范（2003）》、《医疗器械临床试验规定（2004）》、WMA《赫尔辛基宣言》和 CIOMS《人体生物医学研究国际道德指南》的伦理原则，经本伦理委员会审查，同意按所批准的临床研究方案、知情同意书、招募材料开展本项研究。

请遵循 GCP 原则、遵循伦理委员会批准的方案开展临床研究，保护受试者的健康与权利。

研究开始前，请申请人完成临床试验注册。

研究过程中若变更主要研究者，对临床研究方案、知情同意书、招募材料等的任何修改，请申请人提交修正案审查申请。

发生严重不良事件，请申请人及时提交严重不良事件报告。

请按照伦理委员会规定的年度/定期跟踪审查频率，申请人在截止日期前 1 个月提交研究进展报告；申办者应当向组长单位伦理委员会提交各中心研究进展的汇总报告；当出现任何可能显著影响试验进行、或增加受试者危险的情况时，请申请人及时向伦理委员会提交书面报告。

研究纳入了不符合纳入标准或符合排除标准的受试者，符合中止试验规定而未让受试者退出研究，给予错误治疗或剂量，给予方案禁止的合并用药等没有遵从方案开展研究的情况；或可能对受试者的权益/健康、以及研究的科学性造成不良影响等违背 GCP 原则的情况，请申办者/监查员/研究者提交违背方案报告。

申请人暂停或提前终止临床研究，请及时提交暂停/终止研究报告。

| | |
|---|---|
| 完成临床研究，请申请人提交结题报告。 | |
| 年度/定期跟踪审查频率 | 12 个月 |
| 有效期 | 自批件下发之日起一年内有效 |
| 联系人与联系电话 | 赵鸿　王华 0731-85369233 |
| 主任委员签字 | [signature] |
| 伦理委员会 | 湖南中医药大学第一附属医院伦理委员会　（盖章） |
| 日期 | 2013 年 1 月 24 日 |

# 湖南中医药大学第一附属医院伦理委员会

# 审查会议签到表

日期：2013年1月23日

伦理委员会到会委员签名：

| 姓名 | 性别 | 专业情况 | 签名 |
|---|---|---|---|
| 郭志华 | 男 | 湖南中医药大学第一附属医院<br>心血管内科　主任医师　教授 | [illegible] |
| 贺菊乔 | 男 | 湖南中医药大学第一附属医院<br>中医外科　主任医师　教授 | [illegible] |
| 赵艳玲 | 女 | 湖南中医药大学第一附属医院<br>主任医师　教授 | [illegible] |
| 陈其华 | 男 | 湖南中医药大学第一附属医院<br>中医外科　主任医师　教授 | [illegible] |
| 黄孟君 | 男 | 湖南中医药大学第一附属医院<br>中医消化　教授 | [illegible] |
| 张月娟 | 女 | 湖南中医药大学第一附属医院<br>护理　主任护师　教授 | [illegible] |
| 张志国 | 男 | 湖南中医药大学第一附属医院<br>药学　主任药师　教授 | 张志国 |
| 谭劲 | 男 | 湖南中医药大学第一附属医院<br>中西医结合口腔　主任医师　教授 | 谭劲 |
| 谢海波 | 男 | 湖南中医药大学第一附属医院<br>中医内科　副主任医师　副教授 | [illegible] |
| 管小平 | 男 | 律师，融源律师事务所 | [illegible] |
| 钟晓 | 女 | 保险<br>太平人寿湖南分公司，业务经理 | 钟晓 |
| 赵鸿（秘书） | 女 | 湖南中医药大学第一附属医院<br>护理　副主任护师　副教授 | [illegible] |

**Attachment 8: Ethical approval of Hengyang Hospital affiliated to Hunan University of Chinese Medicine**

湖南中医药大学附属衡阳医院伦理委员会文件（EC-AF-2013001）

## 伦理审查批件

| 项目名称 | 电针治疗女性单纯性压力性尿失禁有效性和安全性多中心随机对照试验 | | |
|---|---|---|---|
| 项目编号 | 2013EC001 | 项目来源 | “十二五”国家科技支撑计划 |
| 牵头单位 | 湖南中医药大学附属衡阳医院 | | |
| 申办者（如有） | | | |
| 主要研究者 | 岳增辉 | | |
| 审查类别 | 初始审查 | 审查方式 | 会议审查 |
| 审查日期 | 2013.2.17 | 审查地点 | 医院门诊楼 11 楼会议室 |
| 审查委员 | 王诚喜、龙双才、邹岳萍、徐基平、钟新林、匡肇、董秋萍、贺新民、谢军、万贤明、谢春亮 | | |
| 批准文件 | 研究方案：（VERSION 1.0_20121105），知情同意书（V1.0） | | |
| 审查意见 | 根据卫生部《涉及人的生物医学研究伦理审查办法（试行）》、国家药品食品监督管理局《药物临床试验质量管理规范》、《医疗器械临床试验规定》、《药物临床试验伦理审查工作指导原则》、国家中医药管理局《中医药临床研究伦理审查管理规范》以及《赫尔辛基宣言》和国际医学科学组织委员会颁布的《人体生物医学研究国际道德指南》的伦理原则，经本伦理委员会审查，同意按所批准的临床研究方案、知情同意书、招募材料开展本项研究。<br>请遵循 GCP 原则、遵循伦理委员会批准的方案开展临床研究，保护受试者的健康与权利。<br>研究过程中若变更主要研究者，对临床研究方案、知情同意书、招募材料等的任何修改，请申请人提交修正案审查申请。<br>如发生严重不良事件以及影响研究风险受益比的非预期不良事件，请申请人及时提交严重不良事件报告。<br>请按照伦理委员会规定的年度/定期跟踪审查频率，申请人在截止日期前 1 个月提交研究进展报告；申报者应当向组长单位伦理委员会提交各中心的研究进展的汇总报告；当出现任何可能显著影响试验进行或增加受试者危险的情况时，请申请人及时向伦理委员会提交书面报告。<br>研究纳入了不符合纳入标准或符合排除标准的受试者，符合中止试验规定而未让受试者退出研究，给予错误治疗或剂量，给予方案禁止的合并用药等没有遵从方案开展研究的情况；或可能对受试者的权益/健康以及研究的科学性造成不良影响等违背 GCP 原则的情况，请申办者/监察员/研究者提交违背方案报告。<br>提前终止或完成临床研究，请及时提交结题报告。 | | |
| 有效期 | 2012 年 12 月 20 日~2013 年 12 月 19 日 | | |
| 联系人及电话 | 谢军，0734-8137737 | | |
| 主任委员签字 | [signature] | | |
| 湖南中医药大学附属衡阳医院伦理委员会（盖章） | | | |
| 2013 年 2 月 19 日 | | | |

[Stamp: 湖南中医药大学附属衡阳医院 伦理委员会]

共 1 页/第 1 页

版本号：1.00/版本日期：20130211

**Attachment 9: Ethical approval of Hubei Provincial Hospital of TCM**



湖北省中医院伦理委员会

Ethics Committee of Hubei Province Hospital of Traditional Chinese Medicine

# 伦理审查批件

## Ethics Review Approval

| 批件号 | HBZY2013-C007-01 | | |
|---|---|---|---|
| 项目名称 | 电针治疗女性单纯性压力性尿失禁有效性和安全性多中心随机对照试验 | | |
| 申办者 | 中国中医科学院广安门医院 | | |
| 研究单位 | 中国中医科学院广安门医院、北京中医药大学东直门医院、四川大学华西医院、中国中医科学院西苑医院、湖南中医药大学附属衡阳医院、湖南省中医院、上海中医药大学附属岳阳中西医结合医院、天津中医药大学第一附属医院、陕西省中医医院、江苏省中医院、山西中医学院中西医结合医院、湖北省中医院 | | |
| 主要研究者 | 周仲瑜 主任医师 | | |
| 审查类别 | 初始审查 | 审查方式 | 会议审查 |
| 审查日期 | 2013-01-23 | 审查地点 | 湖北省中医院伦理办会议室 |
| 审查委员 | 涂远超、文建华、刘建忠、郭艳红、费兰波、程业刚、王小琴、高文喜、周忠明、胡晓雪、石艳红、吴胜利 | | |
| 批准文件 | 临床研究方案版本号/日期：VERSION1.0_20121106/2012-11-06；<br>受试者知情同意书版本号/日期：V1.0/2012-11-06。 | | |

**审查意见**

根据卫生部《涉及人的生物医学研究伦理审查办法（试行）》（2007）、SFDA《药物临床试验质量管理规范（2003）》、《医疗器械临床试验规定（2004）》、WMA《赫尔辛基宣言》和CIOMS《人体生物医学研究国际道德指南》的伦理原则，经本伦理委员会审查，同意按所批准的临床研究方案、知情同意书开展该项研究。

请遵循GCP原则、遵循伦理委员会批准的方案开展临床研究，保护受试者的健康与权利。研究开始前，请申请人完成临床试验注册。

研究过程中若变更主要研究者，对临床研究方案、知情同意书、招募材料等的任何修改，请申请人提交修正案审查申请。发生严重不良事件，请申请人及时提交严重不良事件报告。

请按照伦理委员会规定的年度/定期跟踪审查频率，申请人在截止日期前1个月提交研究进展报告。

出现没有遵从方案开展研究的情况；或可能对受试者的权益/健康、以及研究的科学性造成不良影响等违背GCP原则的情况，请申办者/监查员/研究者提交违背方案报告。

申请人暂停或提前终止临床研究，请及时提交暂停/终止研究报告。

完成临床研究，请申请人提交结题报告。

| 跟踪审查频率 | 12个月 |
|---|---|
| 有效期 | 12个月 |
| 联系人与联系电话 | 张馨、陈学军 027-88920956 |
| 主任委员签字 | 涂远超 |

湖北省中医院伦理委员会（盖章）

日期：2013.1.30

湖北省中医院伦理委员会

Ethics Committee of Hubei Province Hospital of Traditional Chinese Medicine

# 会议签到表

Sign-in Sheet of Meeting

| | |
|---|---|
| 项目名称 | ①肾力欣颗粒Ⅱ期临床试验②红花黄色素注射液Ⅱb 期临床试验③ZONCARE-S9 型全数字彩超临床验证④灯盏丹芪胶囊III期临床试验⑤臭氧综合治疗仪临床验证⑥连花急支片III期临床试验⑦电针治疗女性尿失禁⑧扶阳罐疗法⑨龙牡壮骨颗粒临床试验 |
| 会议日期 | 2013 年 1 月 23 日 |

| 姓名 | 性别 | 专业背景 | 签名 |
|---|---|---|---|
| 涂远超 | 男 | 心血管内科 | [illegible] |
| 巴元明 | 男 | 中医肾病 | 请假 |
| 刘建忠 | 男 | 中医儿科 | [illegible] |
| 文建华 | 男 | 中医内科 | [illegible] |
| 郭艳红 | 女 | 行政管理 | [illegible] |
| 费兰波 | 女 | 中医针灸 | [illegible] |
| 程业刚 | 男 | 中西医结合肾病 | [illegible] |
| 王小琴 | 女 | 中医内科 | [illegible] |
| 高文喜 | 男 | 中医外科 | [illegible] |
| 周忠明 | 男 | 妇产科 | [illegible] |
| 胡晓雪 | 女 | 药学 | [illegible] |
| 吴胜利 | 男 | 律师 | [illegible] |
| 石艳红 | 女 | 社区警务 | 石艳红 |
| 张　馨 | 女 | 中西医结合临床 | [illegible] |
| 陈学军 | 男 | 科研管理 | [illegible] |

**Attachment 10: Ethical approval of Jiangsu Province Hospital of TCM**

# 伦理审查批件

| 批件号 | 2013NL-013-04 | | |
|---|---|---|---|
| 项目名称 | 电针治疗女性单纯性压力性尿失禁有效性和安全性多中心随机对照试验 | | |
| 项目来源 | “十二五”国家科技支撑计划 | | |
| 研究单位 | 江苏省中医院，中国中医科学院广安门医院 | | |
| 主要研究者 | 孙建华 | | |
| 审查类别 | 复审申请 | 审查方式 | 快速审查 |
| 审查日期 | 2013 年 06 月 25 日 | 审查地点 | |
| 审查委员 | 殷立平 | | |
| 审查文件 | 复审申请<br>技术合作合同 | | |

**审查意见**

根据卫生部《涉及人的生物医学研究伦理审查办法（试行）》（2007）、SFDA《药物临床试验质量管理规范（2003）》、《医疗器械临床试验规定（2004）》、WMA《赫尔辛基宣言》和 CIOMS《人体生物医学研究国际道德指南》的伦理原则，经本伦理委员会审查，同意按所批准的临床研究方案、知情同意书、招募材料开展本项研究。

请遵循 GCP 原则、遵循伦理委员会批准的方案开展临床研究，保护受试者的健康与权利。研究开始前，请申请人完成临床试验注册。研究过程中若变更主要研究者，对临床研究方案、知情同意书、招募材料等的任何修改，请申请人提交修正案审查申请。发生严重不良事件，请申请人及时提交严重不良事件报告；紧急报告之后，尽快提交详细的严重不良事件随访报告。请按照伦理委员会规定的年度/定期跟踪审查频率，申请人在截止日期前 1 个月提交研究进展报告；申办者应当向组长单位伦理委员会提交各中心研究进展的汇总报告；当出现任何可能显著影响试验进行、或增加受试者危险的情况时，请申请人及时向伦理委员会提交书面报告。研究纳入了不符合纳入标准或符合排除标准的受试者，符合中止试验规定而未让受试者退出研究，给予错误治疗或剂量，给予方案禁止的合并用药等没有遵从方案开展研究的情况；或可能对受试者的权益/健康、以及研究的科学性造成不良影响等违背 GCP 原则的情况，请申办者/监查员/研究者提交违背方案报告。申请人暂停或提前终止临床研究，请及时提交暂停/终止研究报告。完成临床研究，请申请人提交结题报告。本项临床试验应当在批准之日起一年内实施，逾期未实施的，本批件自行废止。

| 年度/定期跟踪审查频率 | 请于 2014 年 06 月 25 日前 1 个月提交研究进展报告 |
|---|---|
| 有效期 | 12 个月 |
| 联系人与联系电话 | 吴静 31618 |
| 主席签字 | [signature] |
| 伦理委员会 | 南京中医药大学附属医院（江苏省中医院）伦理委员会（盖章） |
| 日期 | 2013 年 06 月 25 日 |

南京中医药大学附属医院 伦理委员会

**Attachment 11: Ethical approval of Shanxi Province Hospital of TCM**

## 陕西省中医医院伦理委员会

## 临床研究伦理审查批件

（2013）伦审第（02）号

<table>
<tr><td>项目名称</td><td colspan="6">针灸疗效国际多中心临床评价研究<br>(电针治疗女性单纯性压力性尿失禁有效性和安全性多中心随机对照试验)</td></tr>
<tr><td>申请单位</td><td colspan="6">陕西省中医医院针灸科</td></tr>
<tr><td>项目来源</td><td>“十二五”国家科技支撑计划</td><td>批准文号</td><td colspan="4">课题编号：2012BAI24B01</td></tr>
<tr><td>承担研究任务科室</td><td>针灸科</td><td>主要研究者</td><td>苏同生</td><td>职称</td><td colspan="2">主任医师</td></tr>
<tr><td>会议时间</td><td>2013 年 02 月 01 日</td><td>会议地点</td><td>院会议室</td><td>审 查 方 式</td><td colspan="2">会议审查</td></tr>
<tr><td>审查文件</td><td colspan="6">1、 临床研究方案（版本号：VERSION1.0_20121106）;<br>2、 例报告表(版本号:VERSION1.0_201201109);<br>3、 知情同意书；<br>4、 研究者专业履历及专业科室人员配备、设备设施情况；<br>5、 研究者手册； 有☐ 无■<br>6、 其他伦理委员会对本研究项目的决定； 有■ 无☐</td></tr>
<tr><td>审查内容</td><td colspan="6">研究者的资格： 符合要求■ 不符合要求☐<br>人员配备： 符合要求■ 不符合要求☐<br>设备条件： 符合要求■ 不符合要求☐<br>知情同意书： 符合要求■ 不符合要求☐<br>获取知情同意书的方法：恰当■ 不恰当☐<br>研究方案： 符合要求■ 不符合要求☐<br>受试者因参加临床试验 有有效抢救措施■ 无有效抢救措施☐<br>发生不良反应或意外： 有补偿规定■ 无补偿规定☐</td></tr>
<tr><td rowspan="2">审查意见</td><td>同意</td><td>作必要的修改后同意</td><td>作必要的修正后重审</td><td>不同意</td><td colspan="2">终止或暂停已批准的研究</td></tr>
<tr><td>3 人</td><td>5</td><td>0</td><td>0</td><td colspan="2">0</td></tr>
<tr><td>出席人数</td><td>应到：9 人</td><td>实到：9 人</td><td>回避：1 人(投票时)</td><td colspan="3">请假：0 人</td></tr>
<tr><td colspan="7">审批意见：经审查该项目临床试验方案符合要求,请对知情同意书进行修改，具体为：在“知情同意书.知情告知页”中明确告知受试者，对照组为“安慰对照治疗”。做出上述修改后，同意开展临床研究。</td></tr>
<tr><td colspan="2">主任委员签字：[illegible]<br>日期：2013 年 2 月 1 日</td><td colspan="2">会议记录者签字：[illegible]<br>日期：2013 年 2 月 1 日</td><td colspan="3">联系电话：029-87251691</td></tr>
</table>

# 伦理审查批件

| | | | |
|---|---|---|---|
| 项目名称 | 电针治疗女性单纯性压力性尿失禁有效性和安全性多中心随机对照试验 | | |
| 项目编号 | 2012EC007 | 项目来源 | “十二五”国家科技支撑计划 |
| 牵头单位 | 中国中医科学院广安门医院 | | |
| 申办者（如有） | 山西中医学院中西医结合医院 | | |
| 主要研究者 | 王杰 高素云 赵文兵 | | |
| 审查类别 | 初始审查 | 审查方式 | 会议审查 |
| 审查日期 | 2013.2.25 | 审查地点 | 医院五楼会议室 |
| 审查委员 | 樊东升，王毅东，高继宁，蔺涛，刘红玲，田成瑛，闫荔 | | |
| 批准文件 | 研究方案（VERSION1.0_20121106）,知情同意书(V1.0) | | |
| 审查意见 | 根据卫生部《涉及人的生物医学研究伦理审查办法（执行）》、国家药品食品监督管理局《药物临床试验质量管理规范》、《医疗器械临床试验规定》、《药物临床试验伦理审查工作指导原则》国家中医药管理局《中医药临床研究伦理审查管理规范》以及《赫尔辛集宣言》和国际医学科学组织委员会颁布的《人体生物医学研究国际道德指南》的伦理原则，经本伦理委员会审查，同意按所批准的临床研究方案、知情同意书、招募材料开展本项研究。<br>请遵循 GCP 原则、遵循伦理委员会批准的方案开展临床研究，保护受试者的健康与权利。<br>研究过程中若变更主要研究者，对临床研究方案、知情同意书、招募材料等的任何修改，请申请人提交修正案审查申请。<br>如发生严重不良事件以及影响研究风险收益比的非预期不良事件，请申请人及时提交严重不良事件报告。<br>请按照伦理委员会规定的粘度/定期跟踪审查频率，申请人在截止日期前 1 个月提交研究进展报告；定期向牵头单位伦理委员会提交研究进展的汇总报告。<br>提前终止或完成临床研究，请及时提交结题报告。 | | |
| 有效期 | 2013 年 3 月 1 日——2014 年 2 月 28 日 | | |
| 联系人与联系电话 | 蔺涛 0351-2621527 | | |
| 主任委员签字 | | | |
| 山西中医学院中西医结合医院伦理委员会（医务科代章） | | | |
| 日期：2013 年 02 月 25 日 | | | |
